# Enablers of sexual and reproductive health and rights interventions in low- and middle-income countries. Insights from capacity development projects implemented in 13 countries in Africa and Asia

**DOI:** 10.1080/16549716.2022.2114148

**Published:** 2022-09-26

**Authors:** Gilbert Tumwine, Per-Olof Östergren, Anette Agardh, Pius Okong, Benedict Oppong Asamoah

**Affiliations:** a Social Medicine and Global Health, Department of Clinical Sciences, Lund University, Malmö, Sweden; b Department of Obstetrics and Gynecology, St. Francis Hospital Nsambya, Uganda

**Keywords:** Maria Nilsson, Healthcare practitioners, capacity development, capacity building, SRHR interventions, low- and middle-income countries

## Abstract

**Background:**

The global community has committed to achieving universal access to sexual and reproductive health and rights (SRHR) services, but *how* to do it remains a challenge in many low-income countries. Capacity development is listed as a means of implementation for Agenda 2030. Although it has been a major element in international development cooperation, including SRHR, its effectiveness and circumstances under which it succeeds or fails have limited evidence.

**Objective:**

The study sought to examine whether improvement in team capacity of SRHR practitioners resulted in improved organisational effectiveness and/or improved SRHR outcomes in low-income countries.

**Methods:**

The study involved 99 SRHR interventions implemented in 13 countries from Africa and Asia. Self-reported evaluation data from healthcare practitioners who participated in a capacity development international training programme in SRHR was used. The training was conducted by Lund University in Sweden between 2015 and 2019. Logistic regression models were used to examine the association between improved team capacity, improved organizational effectiveness and improved SRHR outcomes, for all the 99 interventions. Adoption of new SRHR approaches (guidelines and policies), media engagement, support from partner organisations and involvement of stakeholders were assessed as possible confounders.

**Results:**

Improved team capacity, support from partner organisations and media engagement were positively associated with improved organisational effectiveness. Improved team capacity was the strongest predictor of organisational effectiveness even after controlling for other covariates at multivariate analysis. However, adopting new SRHR approaches significantly *reduced* organisational effectiveness. Furthermore, support from partner organisations was positively associated with increased awareness of and demand for SRHR services.

**Conclusions:**

Successful implementation of capacity development interventions requires an enabling environment. In this study, an SRHR training programme aiming at improving team capacity resulted in an improvement in organisational effectiveness. Support from partner organisations and media engagement were key enablers of organisational effectiveness.

## Background

Sexual and reproductive health and rights (SRHR) are essential components of good health and sustainable development [1,2]. Although international policies are rather explicit as to *what* needs to be done to achieve universal access to SRHR services, *how* to do it effectively remains a challenge in many low-income countries [3]. Capacity development has been pursued for decades as a vital approach to influence policy and practice in public health interventions. As a result, it is a major element in international development cooperation [4]. The World Health Organization (WHO) defines capacity development as ‘the […] advancement of knowledge and skills among practitioners, the expansion of support and infrastructure for health promotion in organizations, and the development of cohesiveness and partnerships for health in communities … ’ [5].

Proponents of capacity development believe that some low- and middle-income countries have inadequate capacity development systems and require support, through international cooperation, to strengthen their competencies in planning, implementing, evaluating and sustaining interventions [6]. However, often there are limited resources available for necessary capacity development and for addressing the pertinent community health needs concurrently. As a result, capacity development remains a critical missing link in global efforts to improve population health and eradicate poverty [7,8]. The UN sustainable development-target 17.9 acknowledges the singular importance of capacity development and commits to ‘enhance international support for implementing effective and targeted capacity-building in developing countries … ’ [3].

Although multiple capacity development interventions are implemented in low-income countries annually, the majority are not evaluated and those that are evaluated show limited impact [9–12]. Some interventions succeed in one context but fail in another [13,14]. Synthesis of some of the available evidence suggests that a combination of factors is required to support and nurture capacity development interventions. These include individual capacities in knowledge and skills, collective competencies of groups and organisations to implement mechanisms for monitoring interventions, and system capacity to coordinate and work with multiple actors and stakeholders [8,12,15,16]. The nature of the intervention plus the context in which an intervention is implemented also contributes to the outcomes of different capacity development programmes [11,15,17,18].

The international community seeks pathways to achieve universal access to SRHR services by 2030, yet there is a gap in the knowledge of what approaches are effective. Therefore, there is a need to investigate whether capacity development for healthcare practitioners could be effective in strengthening organisational capacity and accelerate the pace at which universal access to SRHR services can be achieved.

Using the ‘Inputs-Process-Output-Outcome-Impact’ framework of evaluation [19], the current study sought to examine whether improvement in team capacity of healthcare practitioners resulted in improved organisational effectiveness and/or improved SRHR outcomes in targeted interventions in low-income countries. In addition, the study assessed the role of adopting new SRHR approaches (new guidelines and policies), involvement of different stakeholders, partner organisations and the media in SRHR interventions. The findings of this study aim to contribute to the much-needed evidence on whether capacity development is an effective strategy for implementing SRHR interventions in low-income countries.

## Methods

### Study setting

The Swedish International Development Agency Cooperation (Sida) [20] has been supporting an international training programme (ITP) approach to capacity development in low- and middle-income countries for decades. The programme comprises the increment of knowledge and skills for individuals and strengthening of organisational competencies to implement change in different sectors of participating countries. The long-term aim of the ITP approach is to contribute to poverty reduction through the development of effective organisations. Participants in ITP are public servants, civil society organisations and the private sector.

Between 2005 and 2018, Sida commissioned Lund University to conduct an ITP in SRHR in low- and middle-income countries [21]. The aim of this ITP was to contribute to the improvement of the living conditions of the poor through improved access to SRHR services. It was founded on the assumption that developing the capacity of key persons in a healthcare system would positively influence organisational capacity and effectiveness to deliver services [22]. The ITP, described in more detail in earlier publications [23,24], had three specific objectives: increasing awareness of and demand for SRHR services, promoting sexuality education, and increasing access to satisfactory SRHR services, which would be achieved through improved organisational effectiveness. Participants in the programme worked in groups (country teams) to address critical gaps in SRHR service delivery through targeted intervention code named ‘change projects’. Participants were guided to design and implement the interventions following a set of principles derived from project design and implementation framework tools such as the Logical Framework Approach (LFA) and Results-Based management (RBM) [25]. The interventions addressed specific SRHR needs of specified target populations in their health systems. The change project themes included youth-adolescent SRH, maternal health, neonatal health, STI/HIV/AIDS/cervical cancer, sexual and gender-based violence (SGBV), sexual minorities/LGBTQ, and commercial sex workers. The change projects were designed and implemented by the participants with support from different stakeholders, partner organisations and Lund University supervisors. The ITP was implemented through five phases (Table 1). The data used in this study were collected at the end of the fifth phase, that is, 6 months after implementation of change projects.

**Table 1.** Enablers of SRHR interventions in low- and middle-income countries: Phases of the international training programme in sexual and reproductive health and rights (SRHR).

Phase/setting	Activity	Duration
Phase 1/home countries	Participants received updated reference literature about SRHR’s international policies and guidelines.	2 months
Phase 2/Sweden	An advanced training in SRHR was conducted at Lund University using various pedagogical methods. Participants were guided by supervisors to plan and design change projects.	4 weeks
Phase 3/home countries	Participants implemented change projects in consultation with stakeholders.	6 months
Phase 4/Asia or Africa	All participants from the same cohort gathered to present their implementation progress, shared experiences and planned for sustainability at a results seminar.	1 week
Phase 5/home countries	Using feedback from phase 4, participants finalised their change projects, presented their final reports to key stakeholders and completed an evaluation questionnaire.	6 months

### Study population

The study focuses on change projects as the study population. Change project teams consisted of 3–6 healthcare practitioners from the same country. The projects examined in this study were implemented between the years 2015 and 2019 in Bangladesh, Ethiopia, Uganda, Tanzania, Zimbabwe, Kenya, India, Myanmar, South Sudan, Liberia, Zambia, Cambodia, and Sudan. The healthcare practitioners were of different gender and professional backgrounds, working in various sectors in their respective healthcare systems. They included teachers, nurses, midwives, doctors, managers, and policymakers.

### Study design

Retrospective quantitative data collected using a structured questionnaire consisting of 36–48 items were obtained. The questionnaire was designed by the ITP, and the data were collected as part of ITP outcome evaluation. In the questionnaire, participants reported on different aspects of ITP and their change projects. The data utilised for this study represent participants’ self-reported evaluation of the effects of change projects on organisational effectiveness and SRHR outcomes among target populations and their perceived role of different stakeholders, partner organisations and the media during the implementation of change projects.

### Procedure

The data which was collected by the ITP and stored in a secure database at Lund University were retrieved, entered into SPSS software, cleaned and re-coded. One questionnaire was found empty and excluded from the study. Using each participant’s country name at registration, ITP intake number, and change project theme, participants’ responses were linked with their respective change projects. For each variable, a team response was then obtained as the sum of individual responses divided by the number of individuals in each change project, i.e. an average score of each variable was obtained as a group response.

### Description of the conceptual model

**Figure 1.**
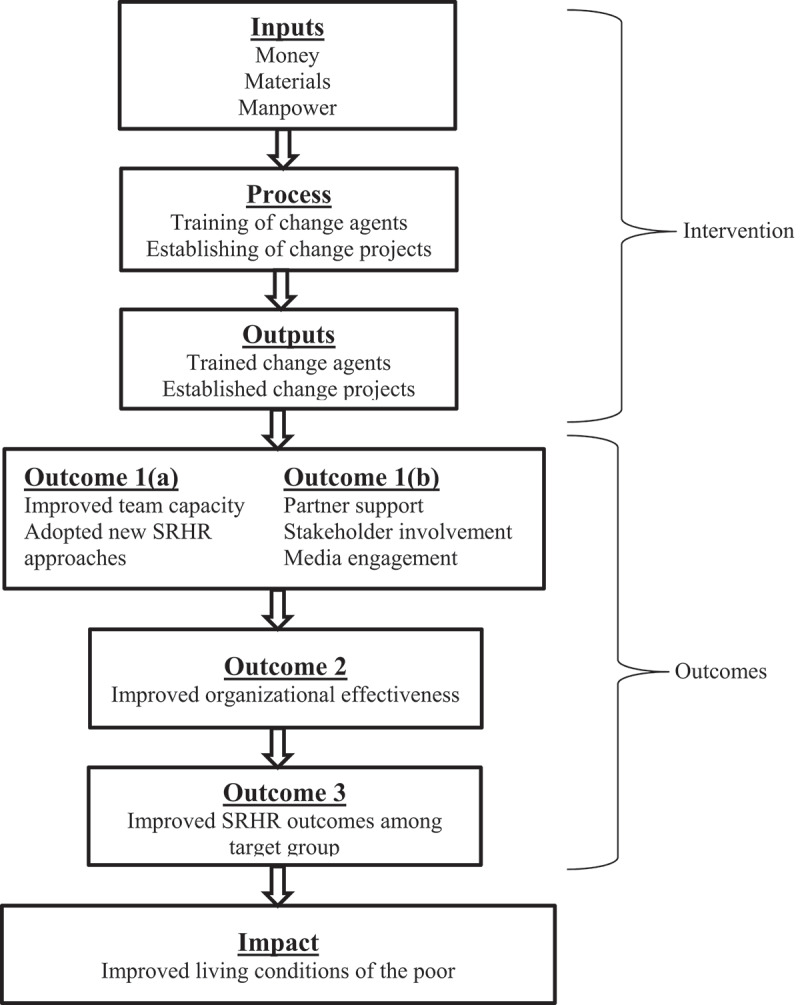
The ‘Inputs-Process-Outputs-Outcomes-Impact’ framework used in the evaluation of capacity development interventions implemented in 13 countries in Africa and Asia (*N* = 99).

Figure 1 illustrates the conceptual relationship between the different components of the ITP intervention, examined using the ‘Inputs-Process-Output-Outcome-Impact’ framework of evaluation [19]. The inputs were the resources used during the training programme including money, materials, and manpower. The processes included the training activities and interactions with stakeholders, partner organisations and target groups, which culminated in the change project outputs. The outputs were the trained ‘change agents’ and the ‘blueprints’ for change projects.

Change project outcomes were categorised (for each change project) in terms of three outcomes. Outcome 1a and Outcome 1b consisted of self-reported changes in group performance after the training including ‘improved team capacity’, ‘adopted new SRHR approaches’, secured ‘partner support’, ‘stakeholder involvement’ and ‘media engagement’ in change project activities. Outcomes 2 and 3 represented the extent to which the change projects achieved ‘Organisational effectiveness’ and ‘improved SRHR outcomes’, respectively, among the target groups.

### Definition of variables

There were 36–48 items in the questionnaire. Factor analysis was used to reduce the large number of variables into fewer variables that were related by the underlying latent meaning. Items were grouped together if the item had a factor loading of at least 0.4 (Varimax rotation).

### Independent variables

The ITP in SRHR aimed at improving team capacity to influence organisational change and improve SRHR outcomes. Hence, this study examined ‘improved team capacity’ (having obtained new SRHR knowledge and technical skills) as the main exposure variable. Having ‘adopted new SRHR approaches’, securing ‘partner support’, ‘stakeholder involvement’, and ‘media engagement’ were analysed as covariates.

### Improved team capacity

Improved team capacity was defined as the extent to which the ITP intervention led to acquisition of new SRHR knowledge and technical skills and improved preparedness of teams to implement change. It was assessed from the responses to the following six survey statements: ‘ITP provided new knowledge on the subject matter’, ‘ITP improved my technical skills to plan and implement a change’, ‘ITP provided skills regarding how to deal with the change processes within the organisational framework’, ‘ITP had an important impact on value issues that were important for the implementation of the change’, ‘ITP gave me access to a network of colleagues and other individuals of importance for the change implementation’, and ‘ITP made me “think outside the box” which became an important factor for the change implementation’. The responses to each statement were coded on a Likert scale of 1–5, where 1 = strongly disagree, 2 = disagree, 3 = neither agree nor disagree, 4 = agree, and 5 = strongly agree. Individuals’ responses were aggregated into group responses for each statement. A composite variable for each change project was obtained by the summation of the six scores provided by the team members. The composite score was then dichotomised based on the median. Scores below and equal to the median meant that the team ‘disagreed’ (reference category) that the ITP intervention had improved team capacity, while scores above the median meant that they ‘agreed’.

### Adopted new SRHR approaches

The following three survey questions were used to assess whether the ITP intervention led to the development and use of new methods, policies, and guidelines in SRHR services: ‘Has your change project led to the development of new guidelines?’, ‘Has your change project led to the implementation of new policy in other organisations?’, and ‘Has your change project led to the implementation of new guidelines in other organisations?’. The responses were coded as 1 = Yes and 2 = No. Individual team members’ responses to each question were aggregated into group responses for each change project. To build a composite variable, the group scores for the three questions were summed up. The variable was then dichotomised into ‘Yes’ for scores below and equal to the median and ‘No’ (reference category) for scores higher than the median.

### Partner support

Partners were persons or organisations that had an active role in the ITP intervention. To assess their role, responses to the following survey questions were used:

‘Which critical factors have contributed to the outcome of your change project?’:

‘Support from others in my home organisation’ and ‘Support from course leaders at Lund University’. The responses were coded as 1 = Yes and 2 = No. Individuals’ responses to each question were aggregated into group responses for each change project. A composite variable was obtained by the summation of the group responses to the two questions and then dichotomised into ‘Yes’ for scores below and equal to the median and ‘No’ (reference category) for scores higher than the median.

### Media engagement

Media engagement was assessed from two items in the questionnaire:

Change projects being reported on ‘Radio’ and ‘Media advocacy for the change project’ having been a critical factor that contributed to the outcome of the change project.

The responses for both statements were coded as 1 = Yes and 2 = No. Individuals’ responses were aggregated into group responses for each statement. A composite variable regarding ‘media engagement’ for each change project was obtained by the summation of the individual responses of team members on the two items and then dichotomised into ‘Yes’ for scores below and equal to the median and ‘No’ (reference category) for scores higher than the median.

### Stakeholder involvement

Stakeholders were organisations which had an interest in the ITP intervention. To assess change projects’ engagement with stakeholders, responses to the following survey questions were used: ‘Has your team or any member of your team held an oral presentation regarding the results of your change project for any of the following audiences?’: ‘Embassy of Sweden’ and ‘UN-agency’, and ‘Has your team presented a sustainability plan of your change project to’: ‘Your organisation management’, ‘Embassy of Sweden’, and ‘UN-agency’? ‘Has your team submitted a written report concerning the result of your change project to the ministry of education or health?’. The responses were coded as 1 = Yes and 2 = No. A composite variable for each change project was obtained from the individual responses of the group to the six questions. The composite scores were then dichotomised into ‘Yes’ for scores below equal to the median and ‘No’ (reference category) for scores higher than the median.

## Dependent variables

### Improved organisational effectiveness

This was defined as the extent to which the change projects resulting from the ITP intervention improved the host organisation’s planning processes, monitoring and evaluation, working routines, and organisational attitudes towards the target groups. Responses indicating participants’ level of agreement or disagreement to the following six survey statements were used:

‘I think my organisation’s effectiveness in planning processes has improved’, ‘I think my organisation’s ability to address internal and/or external factors that may affect a planned project in a positive or negative way has improved’, ‘I think my organisation’s effectiveness in regard to monitoring and evaluation of new projects has improved’, ‘I think my organisation’s effectiveness in regard to working routines has improved’, ‘I think my organisation’s ability to increase the target group’s knowledge and demand for SRHR has improved’, and ‘I think my organisations attitudes towards the target group have changed for the better’.

The responses to these statements were coded on a Likert scale of 1–5 where 1 = Strongly disagree, 2 = Disagree, 3 = Neither Agree nor Disagree, 4 = Agree, and 5 = Strongly Agree. Individuals’ responses were aggregated into group responses for each statement. A composite variable ‘organisational effectiveness’ for each change project was obtained by the summation of the individual group members’ responses regarding the six statements. The composite scores were normally distributed; hence, two outcome categories were created based on the mean. Scores above the mean indicated that the teams ‘agree’ and scores up to and equal to the mean indicated that they ‘disagree’ (reference category) to the statement that the change project had resulted in organisational effectiveness.

### Improved SRHR outcomes

The extent to which the change project had improved access to SRHR services for the target group was assessed from the responses to three survey statements:

‘The programme has increased awareness and demand for SRHR services among the target group’, ‘The programme has contributed to the promotion of sexuality education’, and ‘The programme has contributed to increased access to satisfactory SRH services of good quality for youth, women and men’. The responses to each statement were coded on a Likert scale of 1–4. 1 = Yes to a large extent, 2 = Yes to a sufficient extent, 3 = Yes to a small extent, and 4 = No or not at all. Individual group members’ responses were aggregated into scores for each change project for each variable. The aggregated scores were then dichotomised according to the median. Low scores up to and equal to the median were categorised as ‘Yes’ and high scores as ‘No’ (reference category).

### Statistical analysis

SPSS software version 24 was used for analysis. Using exploratory factor analysis [26], variables with similar latent meaning were grouped together. Composite variables were formed from groups of variables that represented similar latent meaning and were correlated.

If a group contained three or more items, Cronbach’s alpha-scales’ reliability test was conducted to determine the internal consistency of the items in the group before constructing composite variables. Variables were included in the scale if item-total correlation was greater than 0.50 and the scales’ reliability was accepted if the Cronbach’s alpha was greater than 0.60 [27]. The composite variables were checked for multicollinearity, and each variable assessed for normality of distribution and then dichotomised. Cross-tabulation was done to gain insights into the relationship between independent and dependent variables. Bivariate logistic regression was used to determine the association (by crude odds ratio [OR] and 95% confidence interval [CI]) between the independent and dependent variables. Multivariate analysis was conducted between the main exposure variable (improved team capacity) and the dependent variables (improved organisational effectiveness and improved SRHR outcomes) and controlled for other study covariates in separate models. Organisational effectiveness was examined as a potential mediating variable between improved team capacity and improved SRHR outcomes. Statistical significance was accepted when the *p* value was less than 0.05. Missing data were excluded (listwise) from cases required for any analysis.

### Ethical considerations

This study utilised aggregate group responses for analysis. The source documents (evaluation questionnaires) were anonymised to the full extent that no person, group of persons or organisations can be identified in the aggregate data used for analysis. In addition, an advisory opinion was sought, and we received approval from the Regional Ethical Review Board in Lund, Sweden, for a waiver of consent, Approval number DNR 2022-01285-01.

## Results

The change projects had three specific objectives: increasing awareness of and demand for SRHR services, promoting sexuality education, and increasing access to satisfactory SRHR services. It was assumed that the objectives would be achieved through improved organisational effectiveness, using healthcare practitioners as change agents. Between the period 2015 and 2019, a total of 313 change agents were trained, and 99 change projects were implemented in 13 countries in Africa and Asia. The majority (45%) of the change projects focused on youth and adolescent SRHR, while 15.1% were about maternal and neonatal health, and 10.1% were focused on sexuality education. Others focused on sexual and gender-based violence (10.1%), HIV/STI/cervical cancer (8.1%), sexual minorities (4.0%), sex workers (3.0%), and other themes (4.0%).

The scales’ reliability test scores for improved organisational effectiveness, improved team capacity, stakeholder involvement, and adopted new SRHR approaches showed very high internal consistency (0.93, 0.97, 0.83 and 0.79, respectively) (Table 2).

**Table 2.** Enablers of SRHR interventions in low- and middle-income countries: Scales’ reliability test scores.

	Item-total correlation	Cronbach’s alpha if item deleted	Scale’s Cronbach’s alpha
**Improved organisational effectiveness (6 items)**			**0.93**
Organisation’s effectiveness in planning processes has improved	0.83	0.92	
Organisation’s ability to address internal and/or external factors affecting a planned project has improved.	0.80	0.92	
Organisation’s effectiveness in monitoring and evaluation of new projects has improved.	0.84	0.92	
Organisation’s effectiveness regarding working routines has improved.	0.84	0.92	
Organisation’s ability to increase the target group’s knowledge and demand for SRHR has improved.	0.79	0.92	
Organisation’s attitudes towards the target group have changed for the better.	0.73	0.93	
**Improved team capacity (6 items)**			**0.97**
ITP provided new knowledge on the subject matter	0.87	0.96	
ITP improved my technical skills to plan and implement change	0.94	0.96	
ITP provided skills to deal with the change processes within the organisational framework	0.91	0.96	
ITP had an important impact on value issues that were important for the implementation of the change	0.85	0.97	
ITP made me ‘think outside the box’ which became an important factor for the change implementation.	0.94	0.96	
ITP gave access to a network of colleagues and other individuals of importance for the change implementation	0.92	0.96	
**Stakeholder involvement (6 items)**			**0.83**
Oral presentation to Embassy of Sweden	0.58	0.81	
Oral presentation to UN	0.68	0.81	
Presentation of sustainability plan to own organisation	0.61	0.81	
Presentation of sustainability plan to Embassy of Sweden	0.62	0.81	
Presentation of sustainability plan to UN	0.58	0.82	
Writing a report to ministry of health and education	0.52	0.82	
**Adopted new SRHR approaches (3 items)**			**0.79**
Change project led to the development of new guidelines	0.58	0.76	
Change project led to implementation of new policy in other organisations	0.52	0.77	
Change project led to implementation of new guidelines in other organizations	0.62	0.76	

Table 3 summarises the distribution of the change projects’ scores on the different self-reported intervention outcome variables. The lowest possible score for the items accessing improved team capacity was 1 and the highest score was 30. The teams’ scores ranged between 6 and 30 scores, and the median team score was 27. The lowest possible score for the items assessing adopting new SRH approaches was 1 and the highest was 6, and the median score was 4.6. Likewise, the lowest possible score for the items measuring improved organisational effectiveness was 1 and the highest was 30, and the mean score for improved organisational effectiveness was 23.7.

**Table 3.** Enablers of SRHR interventions in low- and middle-income countries: Distribution of self-rated scores for the independent and dependent variables (*N* = 99).

	Median score (range)		Number of projects *N* (%)
Independent variables			
Improved team capacity	27 (6–30)	> Median (Agree)	76 (76.8)
		≤Median (Disagree)	23 (23.2)
Adopted new SRHR approaches	4.6 (1–6)	≤Median (Yes)	41 (41.4)
		> Median (No)	56 (56.6)
		Missing	2 (2.0)
Stakeholder involvement	8 (1–12)	≤Median (Yes)	52 (52.5)
		> Median (No)	45 (45.5)
		Missing	2 (2.0)
Media engagement	3.1 (1–4)	≤Median (Yes)	50 (50.5)
		> Median (No)	49 (49.5)
Partner support	2.3 (2–4)	≤Median (Yes)	47(47.5)
		> Median (No)	43 (43.4
		Missing	9 (9.1)
Dependent variables			
Organisational effectiveness	Mean 23.7 (SD = 3.9)	>Mean (Agree)	58 (58.6)
		≤ Mean (Disagree)	41 (41.4)
Increased awareness and demand for SRHR services	3.6 (3–4)	≤Median (Yes)	39 (39.4)
		> Median (No)	28 (28.3)
		Missing	32 (32.3)
Promoted sexuality education	3.6 (2.6–4)	≤Median (Yes)	39 (39.4)
		> Median (No)	28 (28.3)
		Missing	32 (32.3)
Increased access to SRHR services	3.6 (2.5–4)	≤Median (Yes)	38 (38.4)
		> Median (No)	29 (29.3)
		Missing	32 (32.3)

Chi-square test of association between study variables indicated that improved team capacity, adopting new SRHR approaches, media engagement and support from partner organisations were statistically significantly associated with improved organisational effectiveness. In addition, support from partner organisations was significantly associated with increased awareness of and demand for SRHR services and increased access to satisfactory SRHR services (Supplementary Material, Table S1 and S2).

Bivariate logistic regression was used to assess the effect size of the independent variables on the dependent outcomes (Table 4). Improving team capacity, media engagement, and partner support were statistically significantly associated with improved organisational effectiveness. Improved team capacity was strongly linked to organisational effectiveness, OR = 6.14 (95% CI 2.15–17.52). Likewise, engagement with the media showed a significant positive association with improved organisational effectiveness, OR = 2.64 (95% CI 1.16–6.02). In addition, support from partner organisations was also significantly associated with improved organisational effectiveness, OR = 3.68 (95% CI 1.51–8.97). However, adopting new SRHR approaches showed a negative association with organisational effectiveness, OR = 0.41 (95% CI 0.18–0.94). Support from partner organisations was the only variable found to have a statistically significant positive association with both increased awareness of and demand for SRHR services and increased access to SRHR services among target groups, OR = 3.00 (95% CI 1.05–8.55) and 3.41 (95% CI 1.19–9.76), respectively. No significant association was found between any of the study variables and improved sexuality education.

**Table 4.** Enablers of SRHR interventions in low- and middle-income countries: Bivariate logistic regression between independent and dependent variables (*N* = 99).

	Improved organisational effectiveness	Increased awareness of and demand for SRHR	Promoted sexuality education	Increased access to SRHR services
	OR 95% (CI)	OR 95% (CI)	OR 95% (CI)	OR 95% (CI)
Improved team capacity *Ref (Disagree)*	6.14 (2.15–17.52) ***	0.44 (0.12–1.57)	0.67 (0.19–2.33)	1.08 (0.31–3.84)
Adopted new SRH approaches *Ref (No)*	0.41 (0.18–0.94) *	1.55 (0.55–4.31)	2.69 (0.95–7.64)	0.61 (0.22–1.75)
Stakeholder involvement *Ref (No)*	0.54 (0.24–1.23)	0.91 (0.33–2.52)	0.77 (0.27–2.15)	1.85 (0.67–5.12)
Media engagement *Ref (No)*	2.64 (1.16–6.02) *	1.40 (0.49–3.99)	2.55 (0.84–7.70)	1.53 (0.54–4.37)
Partner support *Ref (No)*	3.68 (1.51–8.97) **	3.00 (1.05–8.55) *	1.96 (0.71–5.41)	3.41 (1.19–9.76) *

*p value ≤0.05, ** *p* value ≤0.01, ***p value ≤0.001.

OR : odds ratio.

When improved team capacity was examined as the main independent variable and adjusted for other covariates with multivariate logistic regression, it remained strongly associated with improved organisational effectiveness. In addition, media engagement and support from partner organisations appeared to strongly confound the effect of improved team capacity on improved organisational effectiveness, by increasing the effect size from an unadjusted OR = 6.14 (95% CI 2.15–17.52) to the adjusted OR = 12.96 (95% CI 3.35–50.19) in the final multivariate analysis (Table 5). Furthermore, in the multivariate analysis, support from partner organisations was the only variable that remained statistically significantly associated with improved organisational effectiveness, OR = 4.42 (95% CI 1.45–13.43) (Table 5), increased awareness of and demand for SRHR services, OR = 3.49 (95% CI 1.15–10.59), and increased access to SRHR services, OR = 3.82 (95% CI 1.22–11.97) (Table 6). None of the study variables seemed to have any statistically significant association with promotion of sexuality education both in bivariate and at multivariate logistic regression. In addition, organisational effectiveness was not found to have any significant mediation effect between improved team capacity and improved SRHR outcomes.

**Table 5.** Enablers of SRHR interventions in low- and middle-income countries: Multivariate logistic regression between improved team capacity and improved organisational effectiveness adjusted for other covariates in four models (*N* = 99).

	Improved organisational effectiveness			
	Model 1 AOR (95% CI)	Model 2 AOR (95% CI)	Model 3 AOR (95% CI)	Model 4 AOR (95% CI)
Improved team capacity *Ref (Disagree)*	5.97 (2.01–17.72) ***	6.11 (2.02–18.47) **	10.76 (3.02–38.36) ***	12.96 (3.35–50.19) ***
Adopted new SRH approaches *Ref (No)*	0.38 (0.16–0.93) *	0.36 (0.14–0.92) *	0.29 (0.10–0.79) *	0.26 (0.079–0.83) *
Stakeholder involvement *Ref (No)*		0.80 (0.31–2.06)	1.15 (0.42–3.18)	1.33 (0.40–4.40)
Media engagement *Ref (No)*			5.07 (1.69–15.16) **	5.28 (1.57–17.71) **
Partner support *Ref (No)*				4.42 (1.45–13.43) **

*p* value* ≤0.05, *p* value** ≤0.01, *p* value *** ≤0.001.

AOR : adjusted odds ratio.

Model 1: Adjusted for ‘adopted new SRHR approaches’.

Model 2: Adjusted for ‘adopted new SRHR approaches’ and ‘stakeholder involvement’.

Model 3: Adjusted for ‘adopted new SRHR approaches’, ‘stakeholder involvement’ and ‘media engagement’.

Model 4: Adjusted for ‘adopted new SRHR approaches’, ‘stakeholder involvement’, ‘home organization support‘ and ‘partner support’.

**Table 6.** Enablers of SRHR interventions in low- and middle-income countries: Multivariate logistic regression between improved team capacity and improve SRHR outcomes adjusted for other covariates in four models (*N* = 99).

	Increased awareness of and demand for SRHR services			
	Model 1 AOR (95% CI)	Model 2 AOR (95% CI)	Model 3 AOR (95% CI)	Model 4 AOR (95% CI)
Improved team capacity *Ref (Disagree)*	0.43 (0.12–1.55)	0.45 (0.12–1.63)	0.47 (0.13–1.78)	0.39 (0.09–1.66)
Adopted new SRH approaches *Ref (No)*	1.49 (0.53–4.22)	1.69 (0.57–5.02)	1.66 (0.55–4.98)	2.31 (0.67–7.96)
Stakeholder involvement *Ref (No)*		0.69 (0.23–2.09)	0.69 (0.23–2.09)	0.62 (0.18–2.06)
Media engagement *Ref (No)*			1.15 (0.37–3.58)	0.96 (0.26–3.45)
Partner support *Ref (No)*				3.38 (1.08–10.51) *
	Increased access to SRHR services			
Improved team capacity *Ref (Disagree)*	0.99 (0.27–3.58)	1.09 (0.30–3.98)	1.32 (0.35–5.08)	1.21 (0.31–4.82)
Adopted new SRH approaches *Ref (No)*	0.61 (022–1.75)	0.53 (0.18–1.63)	0.47 (0.15–1.51)	0.46 (0.13–1.68)
Stakeholder involvement *Ref (No)*		1.97 (0.67–5.80)	1.99 (0.67–5.93)	2.38 (0.71–8.03)
Media engagement *Ref (No)*			1.90 (0.60–5.98)	2.01 (0.53–7.65)
Partner support *Ref (No)*				3.82 (1.22–11.97) *

*p* value* ≤0.05,

AOR : adjusted odds ratio.

Model 1: Adjusted for ‘adopted new SRHR approaches’.

Model 2: Adjusted for ‘adopted new SRHR approaches’ and ‘stakeholder involvement’.

Model 3: Adjusted for ‘adopted new SRHR approaches’, ‘stakeholder involvement’ and ‘media engagement’.

Model 4: Adjusted for ‘adopted new SRHR approaches’, ‘stakeholder involvement’, ‘home organisation support‘ and ‘partner support’.

## Discussion

The Inputs-Process-Outputs-Outcomes-Impact framework was used to examine a linked relationship between the proximal and distal outcomes of the ITP in SRHR. Improved team capacity, support from partner organisations and media engagement were positively associated with improved organisational effectiveness. Improved team capacity was the strongest predictor of organisational effectiveness even after controlling for other first-order outcomes in the multivariate analysis. However, adopting new SRHR approaches seemed to significantly reduce organisational effectiveness, contrary to our assumptions. Furthermore, support from partner organisations was positively associated with increased awareness of and demand for SRHR services and increased access to SRHR services.

There is consensus among practitioners and policymakers that to obtain meaningful and sustained achievements in health goals, critical improvements must be made not only to develop the capacity of communities to demand quality services but also to develop the capacity of organisations to deliver these services more effectively [28,29]. Inadequate organisational capacity among health-sector actors has also been linked to health systems’ ineffective use of resources and unresponsiveness to population needs [30]. This study builds on this knowledge, and more specifically, regarding SRHR interventions, it highlights the potential and necessity of developing the capacity of members of implementing organisations (providing new knowledge, cultivating necessary skills and values systems, and broadening organisational networks) to improve organisational effectiveness and improve SRHR outcomes in low-income countries.

The association between improved capacity of practitioners and improved organisational effectiveness has also been reported in other capacity development-related interventions in healthcare systems. For example, Prashanth et al. [31] showed that improvements in health leaders’ capacity improved health service delivery in low- and middle-income countries as a result of improved planning processes and strengthened supervision of health services. Sobeck and Agius [32] reported that capacity development interventions resulted in ‘greater awareness of needs and improved management knowledge’ among health managers in not-for-profit organisations. Generally, the improved capacity of health managers has been reported as a significant factor in strengthening health services, which in turn translates into an overall improvement in health outcomes [33].

In contrast, adopting new SRHR approaches was statistically significantly associated with reduced organisational effectiveness. This is plausible because building capacity of institutions to adopt new approaches may take time and resources away from specific interventional objectives and is dependent upon the ability of the organisations to absorb the new methods, guidelines or policies [34]. The ITP intervention may have provided a relatively limited time to allow for practitioners to adopt new methods, guidelines and policies.

Other key factors positively associated with improved organisational effectiveness were media engagement and support from partner organisations. Media advocacy has been defined as, ‘the strategic use of mass media to advance public policy initiatives […]’ [35], and if used effectively can increase knowledge among a population and influence policy [36]. Strategic engagement with the media is a well-established strategy to focus public attention on health issues and enhance the involvement of both policymakers and communities [37], with a potential to stimulate and enhance development [38] when appropriately used for agenda-setting [39,40]. SRHR capacity development interventions can harness these unique possibilities to engage with the media and discuss sensitive issues of SRHR in low-income countries where media is not subject to censorship. In this study, media engagement did not seem to increase awareness of and demand for SRHR services nor did it improve access to SRHR services. Nevertheless, media engagement is a potentially valuable tool that should be explored in larger studies, given that young people in most low-income countries are increasingly gaining access to Internet-enabled mobile devices.

Support from partner organisations was reported to increase awareness of and demand for, and improved access to, SRHR services in our study. Local participation and ownership of externally sponsored interventions improve chances of interventions surviving beyond the external support [7], and evidence from evaluation studies reinforces the notion that active involvement by host organisations improves effectiveness and increases chances of interventions being sustained in low-income countries [41,42].

None of the study variables were found to have contributed to increased promotion of sexuality education despite this being one of the specific objectives of the change projects. This is possibly because sexuality is still a taboo in many societies, and discourse on promotion of sexuality education, commonly framed within socio-cultural and religious confines, can be complex. As a result, it is often difficult to reach consensus on SRHR issues such as safe abortion, contraception or sexual orientation [43].

### Methodological considerations

The main strength of the study is that the study population originated from diverse socio-cultural settings in 13 countries from Africa and Asia. Although the findings may not be wholly generalisable to all countries in Africa and Asia, they provide insights on potential enablers of capacity development for SRHR interventions in low-income countries and could be of great value in generating hypotheses for future research in capacity development for SRHR interventions in similar settings. In addition, this study made use of the Inputs-Process-Outputs-Outcomes-Impact framework of evaluation [19]. Although this framework has not been used extensively in evaluation research, the underlying principles have been adopted in the Logical Framework Approach (LFA) and the Results-Based Management (RBM) framework and found to be valuable integral components of project monitoring and evaluation [25,44]. Furthermore, the use of the change projects as unit of analysis distinguishes this study from most evaluation studies that have examined determinants of organisational change in capacity development using individual-level data. The aggregation of individual data into group data provides a more accurate reflection of the evaluation by reducing biases due to differences between individual evaluations. This is particularly important when interventions are implemented by groups, but the evaluation feedback is provided by individuals, which was the case in the ITP intervention [45].

One important limitation of this study is its small sample size. A small sample size increases the risk of type 2 error, and the study could have failed to demonstrate significant differences in outcomes where in fact such differences truly exist. In addition, due to the retrospective nature of the data, the study was prone to recall bias and misclassification which could bias the estimated effects in any direction. Therefore, the conclusions drawn from this study should be interpreted with appropriate caution and need to be replicated in larger prospective studies.

The ITP in SRHR was externally supported by Sida through Lund University. It is possible that participants could have over-reported or under-reported their responses or provided responses perceived to be desirable to either Lund University or Sida. This form of social desirability bias would result in misclassification most likely of non-differential type, which may attenuate some of the true associations found [46]. However, in this study, we believe that social desirability bias was less likely since the questionnaire was self-administered, electronically delivered, and anonymously reported. In addition, participants were assured that their responses could not be linked to them as individuals [47].

The study utilises participants’ self-reported evaluation data. Self-reported evaluation data allows us to understand the intervention from the implementers’ perspective only. The findings could be strengthened by triangulation using perspectives from the target populations, stakeholders, and partners. In addition, due to limited data on some variables, it was not possible to control for a very large range of confounders; for example, the study did not account for the extent to which access to local financing was a factor in determining change project outcomes since no direct funding was provided to implement the change projects. Access to financing could have been an important enabler or hindrance in some contexts. Finally, it was assumed that the enablers of capacity development would be similar across all the SRHR themes. This needs to be examined further in larger studies.

### Conclusion

Successful implementation of capacity development interventions requires an enabling environment. In this study, an SRHR intervention aiming at improving team capacity resulted in improvement in organisational effectiveness. Support from partner organisations and media engagement were also enablers of organisational effectiveness.

## Paper context

Capacity development is listed as a means of implementation for Agenda 2030. However, evidence of its effectiveness and circumstances under which it succeeds or fails is limited. In this study, improving team capacity of healthcare practitioners resulted in improved organisational effectiveness but did not improve access to SRHR services in targeted populations. Instead, support from partner organisations strongly predicted improved SRHR services, highlighting the importance of local ownership of externally funded capacity development interventions.

## References

[1] Starrs AM, Ezeh AC, Barker G, et al. Accelerate progress—sexual and reproductive health and rights for all: report of the Guttmacher–Lancet Commission. Lancet. 2018;391:2642–12. 29753597 10.1016/S0140-6736(18)30293-9

[2] Ghebreyesus TA, Kanem N. Defining sexual and reproductive health and rights for all. Lancet (London, England). 2018; Epub 2018/05/14. PubMed PMID: 29753598 391, 2583–2585. 10.1016/S0140-6736(18)30901-2 29753598

[3] United Nations General Assembly. Transforming our world: the 2030 Agenda for sustainable development. New York, United Nations. 2015. p. A/RES/70/1.

[4] DeCorby-Watson K, Mensah G, Bergeron K, et al. Effectiveness of capacity building interventions relevant to public health practice: a systematic review. BMC Public Health. 2018;18:684. 29859075 10.1186/s12889-018-5591-6 PMC5984748

[5] Smith BJ, Tang KC, Nutbeam D. WHO Health Promotion Glossary: new terms. Health Promot Int. 2006;21:340–345. Epub 2006/09/12. doi: 10.1093/heapro/dal033 PubMed PMID: 16963461. 16963461

[6] United Nations Development Programme (UNDP). Capacity for development: new solutions to old problems. Int J Sustainability in Higher Educ. 2002;3. doi: 10.1108/ijshe.2002.24903dae.008

[7] Ado A. Capacity building in developing and emerging countries: from mindset transformation to promoting entrepreneurship and diaspora involvement. Elie Chrysostome, State University of New York. 2019. Springer Edition. Manage Int/Intl Manage/Gestiòn Internacional. 2020;24:213–214.

[8] Management Sciences for Health. Challenges encountered in capacity building. A review of literature and selected tools [Internet]. 2010. [cited on 2022 Apr 4th] Available from: https://reliefweb.int/report/world/challenges-encountered-capacity-building-review-literature-and-selected-tools.

[9] Agheneza Z. Why development projects fail in Cameroon: evidence from Ngie in the NW province of Cameroon. Int J Rural Manag. 2009;5:73–90.

[10] Banerjee AV, Banerjee A, Duflo E. Poor economics: a radical rethinking of the way to fight global poverty. Public Affairs. 2011;2: 252–257.

[11] Diallo A, Thuillier D. The success of international development projects, trust and communication: an African perspective. Int J Project Manage. 2005;23:237–252.

[12] Ika LA. Project management for development in Africa: why projects are failing and what can be done about it. Project Manage J. 2012;43:27–41.

[13] Munk N. The idealist: Jeffrey Sachs and the quest to end poverty: signal. Int J Therapeutic Massage & Bodywork. 2013;6:3–5. 10.3822/ijtmb.v6i3.230 PMC3757231 24000303

[14] Glewwe P, Kremer M, Moulin S. Many children left behind? Textbooks and test scores in Kenya. Am Econ J Appl Econ. 2009;1:112–135.

[15] Ika LA, Donnelly J. Success conditions for international development capacity building projects. Int. J. Project Manage. 2017;35:44–63.

[16] Colarossi L, Dean R, Stevens A, et al. Sexual and reproductive health capacity building for foster care organizations: a systems model. Child Youth Services Rev. 2019;105:104423.

[17] Ajoy D, Louise S, Arnaldo P. Capacity, complexity and consulting: lessons from managing capacity development projects. A Working Paper 344. UK London: Overseas Development Institute, 2012.

[18] Baser H, Morgan PC. Change and performance study report. (ECDPM Discussion Paper 59B). Maastricht: ECDPM. 2008.

[19] Gadomski A, Black R, Mosley H. Constraints to the potential impact of child survival in developing countries. Health Policy Plan. 1990;5:235–245.

[20] Sida. ITP in sexual and reproductive health and rights [Internet]. 2020 [cited Nov 2021 17th]. Available from: https://training.sida.se/ITP/activities/activitydetails_ext.aspx?inapp=1&id=34®ionid=0&cityid=0&fromdate=&todate=.

[21] ITP300. Sexual and reproductive health and rights. SE-105 25 Stockholm, Sweden: Swedish International Development Agency Cooperation and Lund University; 2016.

[22] Sida. Evaluation of Sida’s ITP approach for capacity development final report [Internet]. 2018. [citted 2021 Nov 17th] Available from: www.Sida.se/publications.

[23] Tumwine G, Palmieri J, Larsson M, et al. ‘One-size doesn’t fit all’: understanding healthcare practitioners’ perceptions, attitudes and behaviours towards sexual and reproductive health and rights in low resource settings: an exploratory qualitative study. PloS One. 2020;15:e0234658–e. PubMed PMID: 32584840. 32584840 10.1371/journal.pone.0234658 PMC7316327

[24] Tumwine G, Agardh A, Gummesson C, et al. Predictors of healthcare practitioners’ normative attitudes and practices towards sexual and reproductive health and rights: a cross-sectional study of participants from low-income countries enrolled in a capacity-building program. Glob. Health Action. 2020;13:1829827. 33076795 10.1080/16549716.2020.1829827 PMC7594875

[25] Örtengren K. A guide to Results-Based Management (RBM), efficient project planning with the aid of the Logical Framework Approach (LFA). 2016. [25 August 2022]. Available:https://www.sida.se/en/publications/a-guide-to-results-based-management-rbm-efficient-project-planning-with-the-aid-of-the-logical-framework-approach-lfa

[26] Watkins MW. Exploratory factor analysis: a guide to best practice. J Black Psychol. 2018;44:219–246.

[27] Said T, H EL. Statistical analysis: internal-consistency reliability and construct validity. Int. J. Quant. Qual. Res. Methods. 2018;6:27–38.

[28] Joffres C, Heath S, Farquharson J, et al. Facilitators and challenges to organizational capacity building in heart health promotion. Qual Health Res. 2004;14:39–60. Epub 2004/01/17. doi: 10.1177/1049732303259802. PubMed PMID: 14725175. 14725175

[29] World Health Organisation (WHO). Towards better leadership and management in health: report of an international consultation on strengthening leadership and management in low-income countries, 29 January −1 February, Accra, Ghana. 2007 Jul 13 2021. Report No.

[30] Ireri S, Walshe K, Benson L, et al. A qualitative and quantitative study of medical leadership and management: experiences, competencies, and development needs of doctor managers in the UK. J Manag Marketing Healthc. 2011;4:16–29.

[31] Prashanth NS, Marchal B, Hoeree T, et al. How does capacity building of health managers work? A realist evaluation study protocol. BMJ Open. 2012;2:e000882. 10.1136/bmjopen-2012-000882 PMC3330260 22466036

[32] Sobeck J, Agius E. Organizational capacity building: addressing a research and practice gap. Eval Program Plann. 2007;30:237–246. Epub 2007/08/11. doi: 10.1016/j.evalprogplan.2007.04.003. PubMed PMID: 17689329. 17689329

[33] Newbrander W, Peercy C, Shepherd-Banigan M, et al. A tool for assessing management capacity at the decentralized level in a fragile state. Int J Health Plann Manage. 2012;27:276–294. Epub 2011/10/29. doi: 10.1002/hpm.1108. PubMed PMID: 22034286. 22034286

[34] Koo BH, Perkins DH. Social capability and long-term economic growth. New York: St. Martin’s Press; 1995.

[35] Wallack L. Media advocacy: a strategy for empowering people and communities. J Public Health Policy. 1994;15:420–436. Epub 1994/01/01. PubMed PMID: 7883943. 7883943

[36] Jernigan DH, Wright PA. Media advocacy: lessons from community experiences. J Public Health Policy. 1996;17:306–330. Epub 1996/01/01. PubMed PMID: 8918021. 8918021

[37] Oronje RN, Undie -C-C, Zulu EM, et al. Engaging media in communicating research on sexual and reproductive health and rights in sub-Saharan Africa: experiences and lessons learned. Health Res Policy Syst. 2011;9:S7. 21679388 10.1186/1478-4505-9-S1-S7 PMC3121138

[38] NN NC. Development information content in the African mass media: a study of two Nigerian dailies. Af Media Rev. 1993;7:75–90.

[39] Vincent R. Mistrusted and sensationalist, or important allies for researchers? Examining the barriers to effective health journalism. In: Global forum for health research forum 11. Beijing China: Panos, London, UK; 2007.

[40] Institute of Medicine (US). Committee on assuring the health of the public in the 21st century. The Future of the Public’s Health in the 21st Century. Washington (DC): National Academies Press (US); 2002. 7, Media. [Internet]. cited 2021 Jul 25]. Available from: https://www.ncbi.nlm.nih.gov/books/NBK221224/25057638

[41] United Nations Development Programme (UNDP). Development effectiveness: review of evaluative evidence. New York: United Nations Development Programme; 2001.

[42] World Bank. Assessing aid: what works, what doesn’t, and why? Washington DC: World Bank and Oxford University Press; 1998.

[43] Keogh SC, Stillman M, Awusabo-Asare K, et al. Challenges to implementing national comprehensive sexuality education curricula in low- and middle-income countries: case studies of Ghana, Kenya, Peru and Guatemala. PLOS ONE. 2018;13:e0200513. 29995942 10.1371/journal.pone.0200513 PMC6040779

[44] Cracknell B. Evaluating the effectiveness of the logical framework system in practice. Project Appraisal. 1989;4:163–167.

[45] Cohen AL, Sanborn AN, Shiffrin RM. Model evaluation using grouped or individual data. Psychon Bull Rev. 2008;15:692–712. 18792497 10.3758/pbr.15.4.692

[46] Krumpal I. Determinants of social desirability bias in sensitive surveys: a literature review. Qual Quantity. 2013;47:2025–2047.

[47] Nederhof AJ. Methods of coping with social desirability bias: a review. Eur J Social Psychol. 1985;15:263–280.

